# Subcellular one carbon metabolism in cancer, aging and epigenetics

**DOI:** 10.3389/freae.2024.1451971

**Published:** 2024-07-31

**Authors:** Tiziano Bernasocchi, Raul Mostoslavsky

**Affiliations:** 1 The Krantz Family Center for Cancer Research, The Massachusetts General Hospital Cancer Center and Harvard Medical School, Boston, MA, United States; 2 The Broad Institute of Harvard and MIT, Cambridge, MA, United States

**Keywords:** one carbon metabolism, DNA methylation, histone methylation, SAM, subcellular metabolism, cancer, aging

## Abstract

The crosstalk between metabolism and epigenetics is an emerging field that is gaining importance in different areas such as cancer and aging, where changes in metabolism significantly impacts the cellular epigenome, in turn dictating changes in chromatin as an adaptive mechanism to bring back metabolic homeostasis. A key metabolic pathway influencing an organism’s epigenetic state is one-carbon metabolism (OCM), which includes the folate and methionine cycles. Together, these cycles generate S-adenosylmethionine (SAM), the universal methyl donor essential for DNA and histone methylation. SAM serves as the sole methyl group donor for DNA and histone methyltransferases, making it a crucial metabolite for chromatin modifications. In this review, we will discuss how SAM and its byproduct, S-adenosylhomocysteine (SAH), along with the enzymes and cofactors involved in OCM, may function in the different cellular compartments, particularly in the nucleus, to directly regulate the epigenome in aging and cancer.

## Introduction

The connection between metabolic intermediates and epigenetic regulation has become increasingly evident in recent years. Metabolites serve as crucial signaling molecules, orchestrating cellular responses and adaptability based on the broad range of nutrients absorbed from our diet. Previous works have shown that dietary changes can influence an organism’s epigenetic landscape, with the potential for these modifications to be inherited by future generations (Padmanabhan et al., 2013; Vaiserman and Lushchak, 2021). A compelling example of this is the devastating famine that occurred at the end of World War II in the Netherlands known as the Dutch famine, where thousands suffered from malnutrition. The children of women who were pregnant during this period exhibited an increased incidence of obesity, schizophrenia, and diabetes. Subsequent studies on this population demonstrated that these children inherited epigenetic marks that significantly impacted their lives (Vaiserman and Lushchak, 2021; González-Rodríguez et al., 2023). One of the key reasons for this effect is due to the fact that all the epigenetic modifications in our chromatin are chemical modifications (metabolites), and thus, their availability directly affects chromatin dynamics. Specifically, changes in an organism’s nutrient intake can significantly influence both DNA and histone modifications. For instance, Acetyl-CoA is a pivotal player in acetylation, S-adenosylmethionine (SAM) is the universal donor for methylation reactions, and adenosine triphosphate (ATP) is a fundamental component for phosphorylation (Wei et al., 1999; Rossetto et al., 2012; Etchegaray and Mostoslavsky, 2016; Mentch and Locasale, 2016; Boon et al., 2020; Guertin and Wellen, 2023). Among these, multiple studies have shown that the availability of Acetyl-CoA is critical for chromatin function. In that context, nuclear acetyl-CoA is derived from multiple pathways, including the (ATP)-citrate lyase (ACLY)-dependent conversion of cytosolic citrate (Wellen et al., 2009), the Acyl-CoA synthetase short-chain member 2 (ACSS2)-dependent conversion of acetate (Bulusu et al., 2017), and the generation of acetyl-CoA from pyruvate by the pyruvate dehydrogenase enzyme. Interestingly, all these enzymes can translocate to the nucleus to generate acetyl-CoA *in situ* to support histone acetylation at specific genes. Such an adaptation was key to drive the expression of lysosomal and autophagy genes to support these metabolic pathways in neurons, as well as to support local repair of DNA damage (Sutendra et al., 2014; Bulusu et al., 2017; Li et al., 2017; Sivanand et al., 2017; Li et al., 2021). Parallel studies have shown that nuclear translocation of ACSS2 in hippocampal neurons was key to allowing histone acetylation locally at neuronal genes, a critical switch to drive long-term spatial memory (Mews et al., 2017). These mechanisms were dependent on phosphorylation reactions initiated by signaling cascades, including activation of the AMPK kinase (Li et al., 2017), suggesting that nuclear metabolic demands were coordinated with nutrient supply. In addition to acetyl-CoA, subcellular availability of the central metabolic coenzyme NAD^+^ also appears critical to supporting epigenetic reactions. Beyond its key roles as an electron carrier in mitochondrial respiration and other redox reactions (Luengo et al., 2021), NAD^+^ can also act as a co-factor in deacetylation reactions driven by the sirtuin enzymes, as well as an ADP-ribosylation donor for the PARP enzymes (Finkel et al., 2009). Given their NAD^+^ dependency, sirtuins can link nutritional states to metabolic reprogramming through sensing of NAD^+^ levels (Liu et al., 2012; Martinez-Pastor and Mostoslavsky, 2012). For instance, the histone deacetylase SIRT6 modulates glycolytic metabolism as a silencer of glycolytic genes in response to glucose availability, a role that defines it as a strong tumor suppressor, while SIRT1 is a major inducer of PGC1α-dependent mitochondrial biogenesis under conditions of nutrient stress (Rodgers et al., 2005). Notably, metabolic stress was shown to increase nuclear levels of NAD^+^ specifically through the nuclear localization and activation of the NAD^+^ synthesis enzyme Nicotinamide Phosphoribosyltransferase (NAMPT) (Svoboda et al., 2019). In addition, SIRT6 was shown to directly activate local NAMPT (Sociali et al., 2019) while in parallel regulating the activity of PARP1 as a sensor of DNA damage (Mao et al., 2011), thus balancing NAD^+^ consumption and production. Local NAD synthesis and SIRT1 activation were also shown as key to preventing axonal degeneration (Araki et al., 2004). Another strong evidence of subcellular regulation of NAD levels was determined in studies showing a switch in expression from the nuclear NMNAT1 to the cytoplasmic NMNAT2 to shift NAD + production from the nucleus to the cytoplasm to inhibit PARP1-dependent parylation of adipogenic genes, in turn driving activation of these genes during adipogenesis (Ryu et al., 2018).

The recent discovery of novel histone modifications involving other metabolites, such as lactylation (Zhang et al., 2019), homocysteinylation (Zhang et al., 2018), dopaminylation (Lepack et al., 2020), benzoylation (Huang et al., 2018), serotonylation (Farrelly et al., 2019), O-GlcNAcylation (Chen et al., 2013), succinylation (Xie et al., 2012), and ADP-ribosylation (Messner and Hottiger, 2011), underscores the profound connection between metabolic pathways and cellular epigenetics. A striking example is lactate, which was conventionally viewed as a mere metabolic waste product. Recent studies have unveiled its role in various pathways, including redox balance and serving as an intermediary energy store, mirroring glucose (Rabinowitz and Enerbäck, 2020). Specifically, lactate has been found to have a significant influence on embryonic stem cells, which are predominantly glycolytic; lactylation marks in these cells are associated with active enhancers crucial for the development of the neural crest and presomitic mesoderm, highlighting the reevaluation of lactate from a waste product to a key epigenetic regulator (Martinez-Outschoorn et al., 2011; Merkuri et al., 2024).

Despite its central role as a metabolic node and modulator of methylation reactions on chromatin, the roles of one-carbon metabolism in the different cellular compartments have been less explored. This review will delve into the one-carbon pathway and its pivotal role in cellular methylation processes.

## OCM pathway overview

**FIGURE 1:**
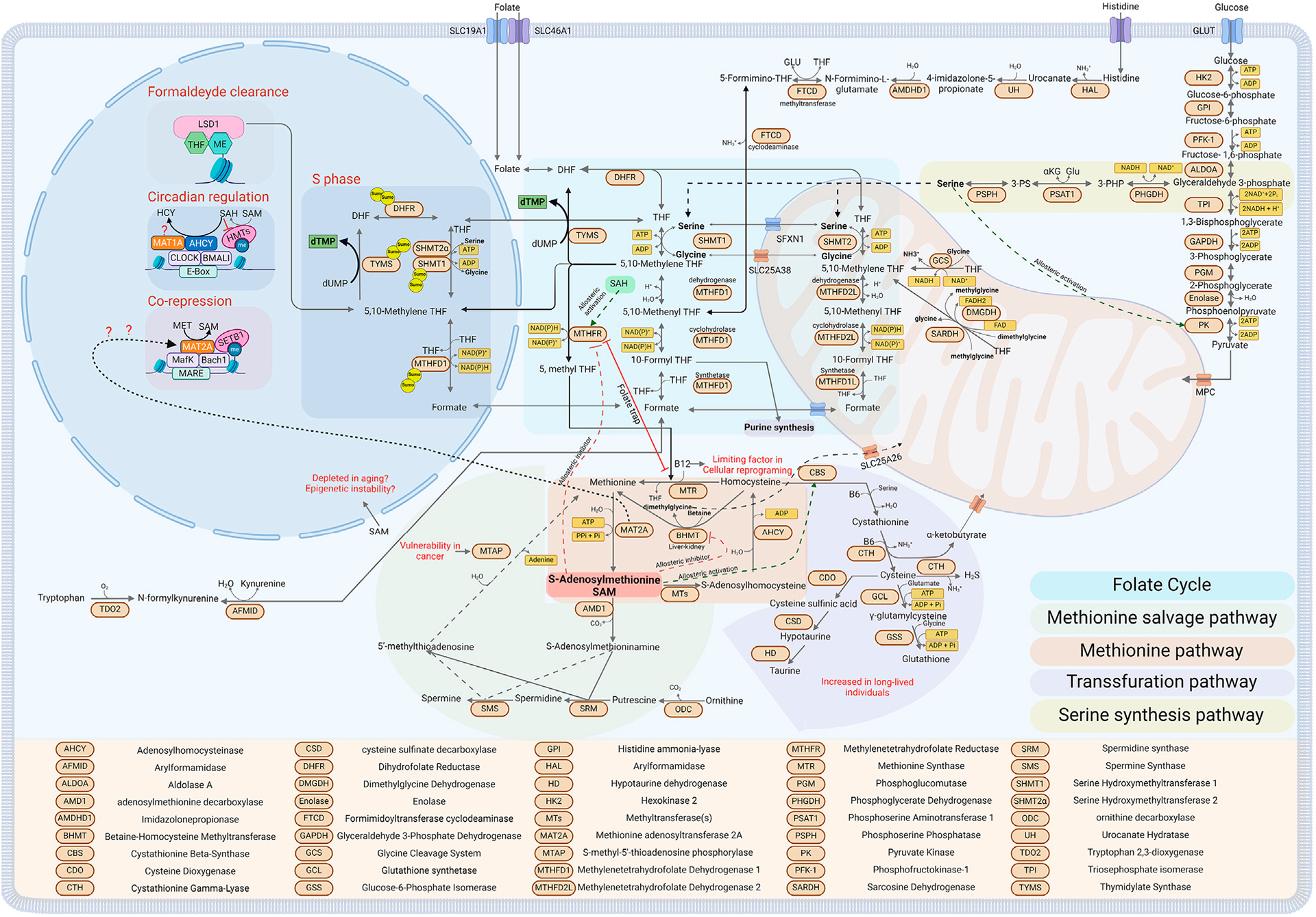
Diagram depicting One Carbon Metabolism and its related metabolic pathways in the different sub-cellular compartments (see text for details). Created with BioRender.com.

One-carbon metabolism, which includes the folate pathway, the methionine cycles, and the transsulfuration pathway, plays a critical role in generating one-carbon units (methyl groups). These groups are required for several metabolic processes, including DNA synthesis, protein methylation (e.g., of histones and creatine), DNA methylation at heterochromatin regions and CpG islands in promoters and enhancers, and the biosynthesis of polyamines and lipids (Yang and Vousden, 2016) (Figure 1).

Starting with the folate cycle, central to one-carbon metabolism, vitamins, and amino acids are utilized as cofactors and methyl group donors, respectively. The cycle is divided between the cytoplasmic and mitochondrial folate cycles (Petrova et al., 2023) (see Figure 1, Folate cycle). In the cytosol, the amino acids serine, histidine, and the metabolite formate act as methyl group donors, with vitamin B9 (folate) serving as the acceptor. Serine is not an essential amino acid; thus, it can be taken up by the cells through different transporters or produced by the cells from other metabolites (Reid et al., 2018; Tajan et al., 2021; Papalazarou et al., 2023). The serine synthesis pathway (SSP) is the major pathway for serine synthesis. The cycle begins with a glycolytic intermediate, 3-phosphoglycerate (3-PG). Initially, Phosphoglycerate dehydrogenase (PHGDH) catalyzes the oxidation of 3-PG to 3-phosphohydroxypyruvate (3-PHP), simultaneously producing NADH. Subsequently, 3-PHP is transformed into 3-phosphoserine (3-PS) by phosphoserine aminotransferase (PSAT1) in a transamination reaction, with glutamate donating the amino group and concurrently generating α-ketoglutarate. The final step is the dephosphorylation of 3-PS to serine, facilitated by phosphoserine phosphatase (PSPH). This reaction produces serine and generates reducing power in the form of NADH and provides α-ketoglutarate, an important intermediate for the TCA cycle (Yang and Vousden, 2016). To underscore the importance of maintaining appropriate serine levels within cells, serine acts as an activator of the enzyme pyruvate kinase M2 (PKM2). This enzyme catalyzes the transfer of a phosphate group to ADP, producing ATP from phosphoenolpyruvate in the final step of the glycolysis pathway, resulting in the formation of pyruvate. Low levels of serine inhibit this reaction, potentially reversing the pathway toward gluconeogenesis, thereby increasing the availability of 3-phosphoglycerate (3-PG) for the serine synthesis pathway (SSP) (Chaneton et al., 2012; Ye et al., 2012) (see Figure 1, Serine synthesis pathway).

Additionally, carbon units for the folate cycle can also be derived from formate, which can donate one carbon directly to tetrahydrofolate in a reversible reaction catalyzed by the tri-enzyme Methylenetetrahydrofolate Dehydrogenase, Cyclohydrolase, and Formyltetrahydrofolate Synthetase 1 (MTHFD1). This reaction consumes an ATP and generates 10-formyl-THF, which can directly enter the *de novo* synthesis of purines or continue through the folate cycle via the cyclohydrolase and dehydrogenase activities of MTHFD1 (Brosnan and Brosnan, 2016) (See Figure 1, Cytoplasmatic Folate cycle). Formate can be generated in cells by tryptophan catabolism or by the activity of aldehyde dehydrogenase class 3 (ADH5), detoxifying formaldehyde from the cells and converting it into the more stable formate (Burgos-Barragan et al., 2017). Moreover, formaldehyde itself can directly condense with THF, driving the formation of 5, 10-methylene THF, the only form of folate that could be combined with homocysteine to generate methionine, which in turn is used by the MAT2 enzymes to synthesize SAM (see Figure 1, Methionine cycle). It has been shown that Lysine-specific demethylase 1 (LSD1) is a folate-binding protein, indicating that while it demethylates and releases the highly reactive metabolite formaldehyde, it could directly recycle it by condensing with THF, thus forming nuclear 5, 10-methylene THF (Luka et al., 2011; Luka et al., 2014; Garcia et al., 2016) (see Figure 1, Formaldehyde clearance). Moreover, histidine catabolism can also generate a carbon unit when it is oxidized to glutamate. The intermediate of the reaction that releases a carbon unit is N-formiminoglutamate (FIGLU) throught the bifunctional enzyme glutamate formiminotransferase (FTCD) that donates one carbon from FIGLU to THF, forming N5-formimino-THF and glutamate. N5-formimino-THF is then further processed to 5, 10-methenyl-THF by formiminotransferase cyclodeaminase (FTCD) (Brosnan and Brosnan, 2020). In this context, histidine catabolism has been suggested as a major consumer of THF, and contributing to the toxicity of the chemotherapeutic antifolate methotrexate (Kanarek et al., 2018). In other organisms but not in humans, the essential amino acid threonine can be converted to Serine in a two-step process: initially, threonine is transformed into 2-amino-3-ketobutyrate by L-threonine dehydrogenase, generating NADH in the process. Subsequently, glycine C-acetyltransferase (GCAT) catalyzes the conversion of 2-amino-3-ketobutyrate to acetyl-CoA and glycine. This pathway has been shown to be essential for mouse Embrionic Stem Cells (mESCs) (Wang et al., 2009; Shyh-Chang et al., 2013).

In the mitochondria, the sources of methyl groups are more diverse. Here, dimethylglycine (DMG), glycine, and sarcosine, along with serine and formate can be utilized as carbon sources to fuel the cycle. The serine required for the mitochondrial folate cycle is transported into the mitochondria through the sideroflexin 1 (SFXN1) transporter (Kory et al., 2018). Once inside, it enters the folate cycle, where it is converted to glycine by serine hydroxymethyltransferase 2 (SHMT2), and a carbon unit is donated to THF to generate 5, 10-methylene THF (Ducker and Rabinowitz, 2017). As highlighted above, most serine is derived from glucose or cellular intake. However, this is not the case in the liver, where it has been shown that Serine hydroxymethyltransferase 2 (SHMT2), which in most organs converts mitochondrial serine to glycine while donating a carbon unit to Tetrahydrofolate, primarily operates in the reverse direction in the liver. This process helps clear the glycine pool and generates serine, which can then be converted by the enzyme serine dehydratase (SDH) into pyruvate and utilized by the TCA cycle (McBride et al., 2024). In the mitochondria, glycine can further contribute to the folate cycle by donating an additional carbon unit through the Glycine Cleavage System (GCS), generating 5, 10-methylene THF, ammonia, and NADH (Kikuchi et al., 2008). Both DMG and methylglycine, also known as sarcosine, can enter the folate cycle. DMG can release two one-carbon units in subsequent reaction steps. First, the enzyme dimethylglycine dehydrogenase (DMGDH) converts DMG to sarcosine, releasing 5, 10-methylene THF and reductive power in FADH2 (Frisell and Mackenzie, 1962). The subsequent reaction utilizes a second mitochondrial enzyme, sarcosine dehydrogenase (SARDH), that catalyzes the oxidation of sarcosine to glycine, generating formaldehyde, which will react with THF to form 5, 10-methylene THF (Wittwer and Wagner, 1981) (See Figure 1, Mitochondria folate cycle).

The methionine cycle is a crucial component of one-carbon metabolism, which is linked to and dependent on the folate cycle. While the folate cycle is essential for generating most of the methyl groups for purine synthesis and reductive power in the mitochondria in the form of FADH2 and NADPH, the methionine cycle’s primary role is generating the universal methyl donor S-adenosylmethionine (SAM), regenerating methionine, and producing polyamines such as spermine and spermidine (Cantoni, 1952; Sanderson et al., 2019) (see, Figure 1, Methionine and salvage cycle). The folate and methionine cycles intersect at the conversion of homocysteine back to methionine by the cytosolic enzyme methionine synthase (MTR), which uses methylcobalamin (methylated vitamin B12) as a cofactor. During this step, a carbon unit generated in the folate cycle and carried by 5-methyl THF is donated to homocysteine, resulting in methionine (Banerjee and Matthews, 1990). This part of the cycle is critical to prevent the accumulation of homocysteine in cells. Dysfunctions led by a polymorphism in MTHFR (the enzyme converting 5, 10-methylene THF to 5-methyl THF) or a lack of vitamin B12 can lead to both hyperhomocysteinemia and folate deficiency. This occurs because 5, 10-methylene THF cannot be recycled in the folate cycle, and homocysteine cannot be recycled in the methionine cycle, thus leading to what is known as the “folate trap” (Scott and Weir, 1981; Gershoni-Baruch et al., 2000; Refsum et al., 2006; Maruti et al., 2009; Hasan et al., 2019) (see Figure 1, Folate trap). The methionine cycle is not the sole pathway that uses homocysteine; the transsulfuration pathway is an alternative route. Here, the enzyme cystathionine β-synthase (CBS) catalyzes the initial reaction, combining serine with homocysteine to generate cystathionine while releasing a molecule of water (Werge et al., 2021). The subsequent reaction, catalyzed by cystathionase, produces cysteine and α-ketobutyrate, releasing ammonia and water. Both reactions require vitamin B6 (pyridoxal phosphate) as a cofactor and can produce hydrogen sulfide as an alternative byproduct. Cysteine then enters the glutathione biosynthesis pathway; initially, it is converted to γ-glutamylcysteine by glutamate-cysteine ligase (GCL), adding glutamate (Chandel, 2021). Subsequently, glutathione synthetase (GSS) adds glycine to form glutathione (GSH), a critical tripeptide that acts as a reducing agent to counteract oxidative stress, to facilitate the detoxification of xenobiotics, and to regulate the cellular redox state (Aquilano et al., 2014) (see Figure 1, Transsulfuration pathway).

The methionine cycle and the transsulfuration pathway are intricately interconnected and regulated by S-adenosylmethionine (SAM) levels. High levels of SAM enhance CBS activity by binding non-covalently to a heme group within CBS, stabilizing the enzyme and increasing the homocysteine flux into the transsulfuration pathway (Prudova et al., 2006). Concurrently, high levels of SAM allosterically inhibit methylenetetrahydrofolate reductase (MTHFR) and betaine-homocysteine S-methyltransferase (BHMT), respectively inhibiting the formation of 5-methyltetrahydrofolate and methionine. Conversely, a low SAM/SAH ratio inhibits CBS due to the lack of SAM and activates MTHFR when bound by S-adenosylhomocysteine (SAH), directing more homocysteine towards the methionine cycle (Kutzbach and Stokstad, 1967; Kutzbach and Stokstad, 1971; Jencks and Mathews, 1987; Ou et al., 2007) (See Figure 1, Allosteric inhibition activation).

Methionine can be regenerated from homocysteine in a folate-independent manner by the enzyme BHMT, which is exclusively expressed in the liver and kidneys. In this pathway, trimethylglycine (known as betaine) donates a methyl group to homocysteine, generating methionine and releasing dimethylglycine (DMG) in an ATP-independent manner (Pajares and Pérez-Sala, 2006). DMG can then enter the mitochondrial folate cycle, as previously mentioned. The subsequent step involves the generation of SAM, facilitated by the methionine adenosyltransferase (MAT) enzyme family. In mammals, there are two MAT enzymes, MAT1A and MAT2A, that encode for two homologous catalytic subunits, α1 and α2, respectively. MAT1A is primarily expressed in the liver and organizes into two isoenzymes, MATIII (a dimer) and MATI (a tetramer) (Kotb et al., 1997; Ramani and Lu, 2017). Conversely, MAT2A is expressed in most tissues and forms the MATII isoenzyme, which can exist as both a dimer and a tetramer (Kotb and Kredich, 1985). MAT enzymes catalyze the reaction between methionine and ATP to generate SAM (also known as AdoMet), releasing phosphate and diphosphate (Niland et al., 2021). At this step, SAM can follow two pathways: it can be used for methylation reactions throughout the cell or enter the polyamine pathway. In the polyamine pathway, SAM is decarboxylated by adenosylmethionine decarboxylase (AMD1) to S-adenosylmethioninamine (dcAdoMet), where it donates a propylamine group to putrescine and spermidine to generate spermidine and subsequently spermine through the enzymes spermidine synthase (SRM) and spermine synthase (SMS), releasing 5-methylthioadenosine (MTA) (Minois et al., 2011). Intriguingly, MTA can reenter the methionine cycle using what is termed the methionine salvage pathway or MTA cycle, where MTA can be recycled back to methionine by S-methyl-5-thioadenosine phosphorylase (MTAP) (Albers, 2009) (See Figure 1, Methionine salvage pathway). The main pathway for SAM involves donating a methyl group in various methylation reactions catalyzed by several methyltransferases, producing S-adenosylhomocysteine (SAH), a potent allosteric inhibitor of many methyltransferases (Richon et al., 2011; Kung et al., 2015). SAH can continue the cycle where it is converted back to homocysteine by S-adenosylhomocysteinase (AHCY) (Turner et al., 1998; Vizán et al., 2021). Moreover, SAM can refuel the folate cycle too by donating a carbon to glycine in the reaction guided by Glycine N-methyltransferase (GNMT) and generating sarcosine that will feed the mitochondrial folate pathway (Yeo and Wagner, 1992; Luka et al., 2009).

## Nuclear OCM and its epigenetics implications

Histone and DNA methylation are metabolic reactions driven by enzymes that add or remove methyl groups (Xiao and Locasale, 2021). These changes depend directly on two key components: the availability of the substrate, S-adenosylmethionine (SAM), and the presence of enzymes. In the case of histones, these enzymes are histone methyltransferases (HMTs), which belong to two main classes: SET domain-containing and non-SET domain-containing enzymes. Both classes require the metabolite SAM as a methyl donor and release S-adenosylhomocysteine (SAH) after the transmethylation reaction (Dillon et al., 2005; Husmann and Gozani, 2019). Interestingly, SAH itself can act as an inhibitor of both classes of HMTs. Therefore, changes in the SAM/SAH ratio can activate or inhibit several methyltransferases, thereby increasing or decreasing histone methylation (Richon et al., 2011; Mentch and Locasale, 2016). This ratio is referred to as the “Methylation Index” because it is directly proportional to the cell’s capacity to methylate (Zhang, 2018) (see Figure 1, Circadian regulation). Mimicking the chemical structure of SAH has led to the development of a new class of drugs targeting methyltransferases (Zhang and Zheng, 2016). A notable example is EZH2, which is highly expressed in several cancers, such as prostate cancer. EZH2 is part of the polycomb repressive complex and represses transcriptional activity through the methylation of H3K27 (Kung et al., 2015; Duan et al., 2020).

As mentioned above, although not strictly related to the subcellular regulation of the OCM, few recent studies have emerged pointing to compartment-specific roles for some enzymes in the cycle (Boon et al., 2020). A significant example is AHCY, which catalyzes the hydrolysis of SAH to homocysteine and has been shown to be associated with CLOCK-BMAL1, the circadian complex that in turn interacts with the SET-dependent MLL family of H3K4 methyl transferases. AHCY’s binding was required to mediate an oscillation of H3K4me3 marks that are circadian dependent, suggesting that these daily chromatin changes are AHCY-dependent and that pharmacological inhibition of AHCY in the hypothalamus alters the amplitude of circadian gene expression. Similarly, another one-carbon pathway gene, MAT1A, was shown to be recruited to the same chromatin complex, suggesting a possible role in the transcriptional regulation of one-carbon enzymes, though its role was not fully explained (Greco et al., 2020) (See Figure 1, Circadian regulation).

Furthermore, MAT2A, which catalyzes the conversion of methionine to SAM, has been shown to interact on a chromatin complex with MafK, Swi/Snf, and NuRD complexes, and its catalytic activity was required for the expression of the MafK-dependent HO-1 gene (Katoh et al., 2011). Subsequent work from the Igarashi lab demonstrated that MAT2A represses the expression of cyclooxygenase 2 (COX2) by specifically interacting with the H3K9 methyltransferase SET-dependent SETB1, inducing the repressive mark H3K9me1/3 and specifically repressing COX-2 genes (Kera et al., 2013). These results suggest a possible compartmentalized role of OCM at the nuclear level (See Figure 1, Co-repression), although it remains unclear whether such roles for MAT2A in the nucleus represent a specific function on few loci or a broader, chromatin-wide role.

In addition, methionine restriction has been shown to impair the methylation pattern of histones. Mentch et al., (2015) demonstrated that H3K4me3, a histone mark commonly found near transcription start site (TSS) regions of actively transcribed genes, was depleted upon methionine restriction. Interestingly, the study found that the methylation mark was depleted across the genome, and the breadth of the peak decreased after methionine restriction, suggesting a potential additional role of these markers as a methyl sink that could be reused in case of necessity. Although Bérénice et al., (2014) linked the breadth of H3K4me3 to transcriptional consistency, this could suggest that in times of need, cells could sacrifice consistency to gain access to additional methyl groups. Along these lines, another study suggested a similar pattern in yeast, demonstrating that demethylation of the Protein Phosphatase 2 A complex (PP2A) in response to methionine deprivation activates PP2A, which then phosphorylates Rph1, a histone demethylase that specifically demethylates H3K4 and H3K36, allowing the preservation of SAM (Ye et al., 2019). Haws et al., (2020) further demonstrated that with the depletion of the cellular SAM pool *in vivo*, cells preferentially conserve H3K9 monomethylation at the expense of di- and tri-methylation, suggesting that maintaining H3K9me1 could help retain the heterochromatin region and preserve genome stability.

## OCM in cancer

The concept of altered metabolic changes in cancer cells was first identified by Otto Warburg in the 1920s, as evidenced by his discovery of increased aerobic glycolysis in cancer cells (Vander Heiden et al., 2009). Additionally, cancer cells, in their quest for rapid proliferation, frequently upregulate several other metabolic pathways, such as the OCM, reflecting the increased demand for synthesizing purine and pyrimidine nucleotides. These nucleotides are essential for synthesizing DNA and RNA, underscoring the critical role of OCM in supporting the proliferative needs of cancer cells (Shuvalov et al., 2017; Petrova et al., 2023). Building on these foundations, anti-folates, targeting the one-carbon pathway, were developed following Sydney Farber’s observation of leukemia’s response to folate-deficient diets (Farber et al., 1948). Despite antifolate’s efficacy in cancer treatment, its broad impact on metabolic pathways can lead to severe side effects.

Traditional strategies targeting cancer metabolism often face limitations due to the essential role of these pathways in normal physiology, leading to limited success. To circumvent these collateral effects, researchers are now exploring the combination of specific diets with targeted therapy. A notable example comes from the work of Gao et al., (2019) who demonstrated that a methionine restriction diet was sufficient to inhibit tumor proliferation *in vivo* in a human PDX model harboring a KRAS mutation. Additionally, the therapeutic effect was enhanced by combining methionine restriction with radiation therapy, which induced a cell-autonomous response dependent on decreased redox and nucleotide metabolism flux. Similarly, a recent study by Li et al., (2024) targeted the one-carbon pathway in liver cancer through the pharmacological inhibition of MAT2A, resulting in increased DNA damage and cell cycle arrest. Further pharmacological screening identified a GSK3 inhibitor that selectively kills MAT2A-inhibited senescent liver cancer cells, suggesting a potential combination therapy between MAT2A and GSK3 inhibitors. Wang et al., (2019) demonstrated that OCM plays a critical role even in tumor-initiating cells (TICs). They performed metabolomic and tracing experiments, specifically identifying an increase in methionine cycle consumption and a high dependency on exogenous methionine in TICs. In this case, pharmacological inhibition of MAT2A was sufficient to cripple the tumor-initiating capability of these cancer cells and alter their epigenetic state. Further highlighting the importance of OCM in cancer, several tumors harbor deletions in the loci containing cyclin-dependent kinase 2A (CDKN2A). Methylthioadenosine (MTAP), as previously discussed, is a gene involved in the methionine salvage pathway and is co-deleted in almost 15% of all cancers with CDKN2A deletion. The deletion of MTAP makes the cells more reliant on the methionine cycle, lacking the ability to recycle methionine from the salvage pathway and, more specifically, depending on MAT2A. Kalev et al., (2021). developed and tested two new inhibitors of MAT2A and demonstrated that cancer cells lacking MTAP were more susceptible to MAT2A inhibitors compared to their wild-type counterparts; they also showed increased sensitivity to taxane therapy *in vivo* when co-treated. Moreover, MAT2A has been identified as a potential target in Diffuse Midline Gliomas (DMGs), a new subclass of high-grade gliomas, over 80% of which are characterized by a hotspot mutation in H3K27 that leads to a global reduction in H3K27me3. Impairment of H3K27me3 spares SAM, increasing its intracellular levels which are used by the RNA methyltransferase METTL16 to methylate the mRNA of MAT2A, causing intron retention and degradation of MAT2A itself. The authors showed that further inhibition of MAT2A activity under these conditions disturbed H3K36me3 methylation and inhibited the oncogenic and developmental transcriptional program of the gliomas, extending survival in multiple models of DMG (Golbourn et al., 2022). The metabolic pathways highlighted above are extremely plastic, exemplified by the carbon units utilized by OCM that can be derived from various sources such as serine, glycine, formate, and others as shown above. For instance, a study by Sullivan et al., (2021) showed that the major source of folate in the cellular environment *in vivo* is 5-methyl-THF, which can sustain the folate cycle *in vivo* instead of folic acid and bypass the efficacy of antifolate therapies such as methotrexate, which inhibit the pathway upstream.

The SSP pathway is tightly regulated in several cancers, underscoring the dependency of cancer cells on serine catabolism. An example is the work by Ding and colleagues, where several cancer cells experiencing serine depletion require the histone 3 lysine 9 (H3K9) methyltransferase G9A to maintain the expression of SSP genes such as PHGDH and SHMT2. Genetic knockdown of G9A inhibited cell proliferation and depleted the serine pool in cancer cells (Ding et al., 2013). Moreover, SHMT2 and GLDC have been shown to antagonize the activity of PKM2 and reduce oxygen consumption in glioblastoma multiforme (GBM), thereby driving a survival advantage by reprogramming the metabolic state of the tumor (Kim et al., 2015). In a similar fashion, cancer cells have been shown to deplete exogenous serine, triggering a p53-dependent activation of the SSP pathway, which suppresses anaerobic glycolysis and increases the flux toward the TCA cycle (Maddocks et al., 2013). The OCM pathway is not only deregulated somatically in cancer. Inherited polymorphisms in the OCM pathway are associated with an increased risk of tumorigenesis. A study exploring the inherited susceptibility to cancer-related epigenetic alterations analyzed 233 patients with colorectal, breast, or lung cancer for germ-line variants in genes critical for methyl group metabolism, including methylenetetrahydrofolate reductase, methionine synthase, and cystathionine β-synthase. This investigation revealed a complex link between genetic predispositions and epigenetic modifications in cancer. Key findings indicated that individuals with the methylenetetrahydrofolate reductase 677 T allele exhibited inherently low genomic 5-methylcytosine levels and less severe global hypomethylation in tumors. Additionally, tumors in patients homozygous for the methionine synthase 2756G allele had fewer hypermethylated CpG islands in tumor suppressor genes, further underscoring the intricate relationship between metabolism and epigenetics in cancer (Paz et al., 2002).

## Impact of OCM on pluripotency and aging

Aging and pluripotency are closely interconnected, albeit in opposing manners. Aging can be described as the gradual exhaustion of an organism’s cellular pluripotency (Pal and Tyler, 2016; López-Otín et al., 2023). Similarly, both processes are heavily influenced by epigenetic modifications. Studies pioneered by the Horvath’s lab defined changes in the methylation of specific CpGs as one of the best predictors of chronological and biological aging in organisms, a phenomenon termed “epigenetic clock” (Horvath, 2013). Conversely, the maintenance of pluripotency necessitates a distinct epigenetic state, as demonstrated by induced pluripotent stem cells (iPSCs), which are reprogrammed through the Yamanaka factors (Oct4, Sox2, Klf4, and c-Myc), which are characterized by a “rejuvenated” epigenetic landscape (Stadtfeld and Hochedlinger, 2010).

The significance of one-carbon metabolism (OCM) in maintaining the pluripotent state of cells was highlighted by the discovery of a more than 200-fold upregulation of threonine dehydrogenase (TDH) in mouse Embrionic Stem Cells (mESCs) (Alexander et al., 2011). Furthermore, the deprivation of threonine among 20 amino acids tested was found critical for the proliferation and differentiation of these cells (Wang et al., 2009). TDH, an enzyme within the OCM, catalyzes the oxidation of threonine to 2-amino-acetate, which subsequently contributes to the generation of acetyl-CoA and glycine through Glycine C-acetyltransferase (GCAT). The depletion of threonine was shown to slow growth and increase differentiation in mESCs due to a reduction in the SAM pool and, consequently, decreasing H3K4me3 markers. Only supplementation with both glycine and acetyl-CoA could rescue this phenotype, suggesting that ES cells require both methyl donors from glycine and reductive power from acetyl-CoA (Shyh-Chang et al., 2013). It is important to note, however, that the TDH enzyme is a pseudogene in human cells and is therefore not active (Edgar, 2002). In another study, Shiraki et al., (2014) tested the reliance of both human Embryonic Stem Cells (ESCs) and iPSCs on single amino acid deprivation. They highlighted that deprivation of leucine, lysine, or methionine inhibited cell proliferation, with methionine deprivation causing the most significant decrease. Similar to previous studies, methionine deprivation led to a decrease in the cellular content of SAM, triggering demethylation of H3K4me3 followed by broader global demethylation, which consequently increased p53 signaling and decreased the expression of the pluripotency marker NANOG.

The reprogramming of iPSCs driven by Yamanaka factors requires extensive epigenetic remodeling, necessitating a large supply of methyl donor groups in the form of SAM. Kovatcheva et al., (2023) demonstrated that during reprogramming, cells deplete the essential cofactor vitamin B12, crucial for methionine and, consequently, SAM production. Replenishing the vitamin B12 pool significantly enhanced the efficiency of reprogramming and prevented illegitimate transcription initiation by maintaining the histone mark H3K36me3. Contrary to the works discussed above suggesting the requirement of one-carbon metabolism (OCM) in pluripotency, overall aging, and lifespan have been associated with what seems to be a downregulation of OCM through methionine restriction (MR). Several studies have shown that dietary restriction of methionine alone is sufficient to improve metabolic function and life extension (Orentreich et al., 1993; Miller et al., 2005; Lees et al., 2014). For example, a study using male Fischer 344 rats showed a 30% increase in lifespan. In a subsequent study on the same rats, it was demonstrated that those on a methionine-restricted diet exhibited reduced visceral fat, decreased levels of insulin and glucose, and increased energy expenditure (DEE) (Malloy et al., 2006). It is important to note that DEE is normalized to body weight, and the MR rats weighed almost half as much as the control group. This further suggests that the lifespan extension phenotype could be attributed to the effects of a restricted caloric diet, which is known to extend lifespan and inhibit full development (McCay et al., 1939; Fontana et al., 2010). In a similar study with *Drosophila*, methionine was found to be necessary and sufficient to increase fecundity in the context of dietary restriction (DR), without negating the positive lifespan effects induced by DR. (Grandison et al., 2009). Moreover, alteration in the OCM has been shown to be in part responsible for the increased life span induced by Metformin, an oral antihyperglycemic drug for type 2 diabetes (T2D). Cabreiro et al., (2013) demonstrated that *C. elegans* co-cultured with *Escherichia coli* exhibited impairment in folate and methionine pathways upon metformin treatment, mimicking a methionine-restricted diet. Mechanistically, metformin inhibits methionine synthase in *E. coli*, causing an accumulation of S-adenosylmethionine (SAM) and 5-methyl tetrahydrofolate (5-methyl THF). This accumulation leads to the inhibition of *C. elegans* SAMS-1, decreasing SAM and S-adenosylhomocysteine (SAH) levels, which drives the life extension phenotype. The study suggests an important role of the microbiota in the influence of the OCM and that the beneficial effect of metformin could be mediated through the OCM pathway.

All the studies above suggest that MR and OCM downregulation are possible mechanisms of healthy and extended life. MR and DR have been shown to increase the fluxes towards the transsulfuration pathway responsible for the production of Hydrogen Sulfide and Taurine (Kabil et al., 2011; Kosakamoto et al., 2023). In line with this, several studies have shown that the aging population has a decrease in the levels of taurine, a semi-essential amino acid that can be taken from the diet or generated by a branch of the OCM, the transsulfuration pathway (See Figure 1, Transsulfuration pathway) (Stuerenburg et al., 2006; Wallace and Dawson, 2009; Ripps and Shen, 2012; Yoshimura et al., 2021; Singh et al., 2023). Taurine deficiency in early life is associated with skeletal, central nervous system and vision impairment (Ripps and Shen, 2012). In a recent study, Singh et al., (2023) demonstrated that taurine levels drop by 80% in the elderly population across several species, including mice, monkeys, and humans. This finding suggests that taurine may play a role in aging-related diseases. To explore this further, the researchers administered daily intraperitoneal taurine to middle-aged mice. The results revealed a significant decrease in cellular senescence, DNA damage, mitochondrial dysfunction, and inflammaging. Notably, both monkeys and mice receiving taurine supplementation exhibited increased health span and lifespan. Moreover, the upregulation of enzymes in the transsulfuration pathway, such as cystathionine β-synthase (CBS), has been observed in long-lived *Drosophila* under dietary restriction (DR), further validating the importance of this pathway and possibly suggesting that one-carbon metabolism (OCM) could be one of the main players in the effects of DR on longevity (Kabil et al., 2011). Similarly, in humans, when comparing taurine and one-carbon metabolism (OCM) levels between centenarians and the normal elderly population, centenarians exhibit upregulation of the transsulfuration pathway and taurine levels and of a microbiome supporting sulfate metabolism (Mota-Martorell et al., 2021; Johansen et al., 2023). These findings suggest that OCM may be crucial for maintaining a healthy aging phenotype.

Changes in DNA and histone methylation are hallmarks of aging (López-Otín et al., 2023). Recently, it has been shown that double-stranded DNA breaks induce the recruitment of DNA repair genes, including chromatin remodeling enzymes such as SIRT6, HDAC1, and PARP1, to repair the breaks. This recruitment can lead to the loss of epigenetic marks, resulting in the loss of a youthful epigenome and an increased rate of aging (Mostoslavsky et al., 2006; Toiber et al., 2013; Tian et al., 2019; Kovatcheva et al., 2023; Yang et al., 2023). The addition or removal of epigenetic marks, as discussed above, are driven by enzymatic reactions, as exemplified by the one-carbon metabolism (OCM) pathway with S-adenosylmethionine (SAM) as the universal methyl donor. Impairment in the OCM pathway could lead to a loss of these epigenetic marks and potentially increase the rate of aging. An interesting example comes from the work of Kang et al., (2024) where similar results were observed *in vivo* in a study comparing aged versus young mice, focusing on the quantity of muscle stem cells (MuSCs). The study revealed that aged MuSCs were associated with decreased levels of heterochromatin, such as H3K9 di- and tri-methylation, and the heterochromatin protein HP1. This reduction was due to the depletion of SAM, which was preferentially utilized for polyamine production at the expense of nuclear methylation. Supplementation with SAM or inhibition of the polyamine pathway greatly enhanced heterochromatin formation, improved the functionality of MuSCs, and reversed the aging phenotype.

## Conclusion

Since the discovery of the double helix and particularly after the Human Genome Project, genetics was hailed as the holy grail for understanding biological processes and curing diseases such as cancer. However, the reality is that while the genome provides the instructions for building cellular components, it does not dictate their use. Often, these decisions are influenced by the environment, which includes metabolites among other factors. Two recent studies challenge this conventional view. The first, by Kong et al., (2024) revisits Knudson’s “two-hit” paradigm in cancer, which posits that both copies of an autosomal tumor suppressor gene must be inactivated for carcinogenesis to occur. This study elegantly demonstrates that a single metabolite, Methylglyoxal (MGO)—derived nonenzymatically from glyceraldehyde-3-phosphate mainly during glycolysis—can act as an oncogene by transiently inactivating Breast cancer type 2 (BRCA2). This transient inhibition of BRCA2 is sufficient to trigger single-base substitutions (SBSs) and increase genomic instability. The second significant study, by Parreno et al., (2024) shows for the first time that a transient epigenetic event can induce tumorigenesis in the absence of additional stimuli. The authors transiently downregulated the polycomb complex (PRC) in flies, which is responsible for depositing H3K27me3 repressive marks and H2AK118UB, which is important for repressing developmental genes through cellular memory. This downregulation activates JAK-STAT signaling and zfh1 (ZEB1 in mammals), which alone was sufficient to induce cancer without driver mutations. Notably, the activity of the PRC complex, including the histone methyltransferase EZH2, as previously discussed, depends on the availability of SAM and is inhibited by SAH. This suggests that transient disruptions in one-carbon metabolism (OCM) could lead to enduring changes in chromatin architecture and, subsequently, cancer.

A longstanding question in oncology is why most cancers develop later in life. One possible explanation is metabolic impairment, which may drive epigenetic changes in chromatin that are sufficient to initiate tumorigenesis independently or to induce DNA damage, thereby leading to cancer-driver mutations. Although we lack current data to support this hypothesis, further studies could establish such a connection. In this context, it might be worthwhile to consider integrating metabolic analysis with standard tests such as PSA or PAP tests.

In this review we aimed to highlight the nuclear role of OCM metabolic enzymes not only as agents in oxidative or reductive reactions but also as signaling proteins. As mentioned above, components of the OCM can relocate to the nucleus, bind to nuclear proteins, and perform signaling functions. This suggests that cellular functions, although compartmentalized, are tightly regulated by metabolic processes, and there is a need to adopt a systems biology approach rather than focusing solely on individual genes or metabolic pathways independently. In discussing the role of OCM in cancer and aging, we highlight the fact that genetic manipulation of the genome, without considering the actuating role of metabolites, could yield modest results, whether in activating pluripotency pathways or in attempts to modulate organismal aging. Future research will undoubtedly continue to elucidate this critical interplay between metabolite levels, metabolic enzymes, subcellular localization, and changes in the chromatin landscape.
